# Mechanical Perspective on Increasing Brush Cytology Yield

**DOI:** 10.1021/acsbiomaterials.3c00935

**Published:** 2024-02-19

**Authors:** Iyad Khamaysi, Ronen Firman, Patrick Martin, Gleb Vasilyev, Evgeniy Boyko, Eyal Zussman

**Affiliations:** † Department of Gastroenterology, The Ruth and Bruce Rappaport Faculty of Medicine, Technion—Israel Institute of Technology, Haifa 3525433, Israel; ‡ Gastroenterology Institute, Rambam Health Care Campus, Haifa 3109601, Israel; § Faculty of Mechanical Engineering, Technion—Israel Institute of Technology, Haifa 3200003, Israel

**Keywords:** brush cytology, malignancy, mucus rheology, biliary, mechanics

## Abstract

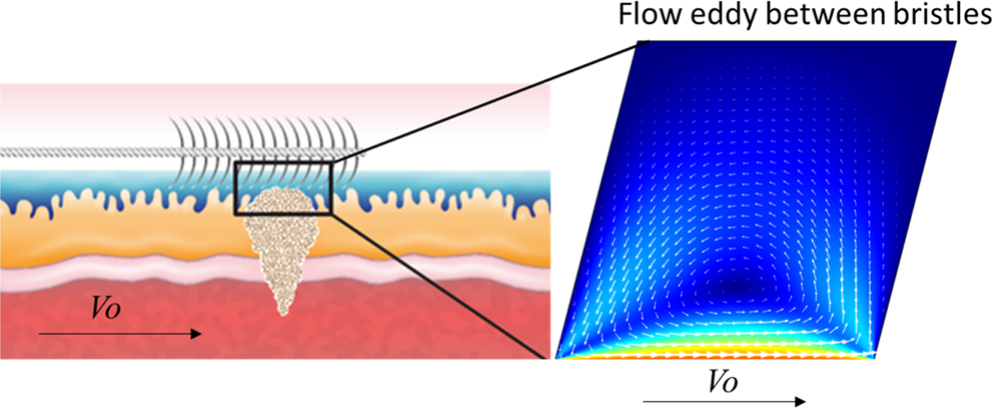

Brush cytology is a sampling technique extensively used for mucosal surfaces, particularly to identify malignancies. A sample is obtained by rubbing the brush bristles over the stricture or lesion several times until cells are trapped. Brush cytology detection rate varies, with malignancy confirmed in 15–65% of cases of adenocarcinoma-associated biliary strictures and 44–80% of cases of cholangiocarcinoma. Despite the widespread use of brush cytology, there is no consensus to date defining the optimal biliary brushing parameters for the collection of suspicious lesions, such as the number of passes, brushing rate, and force applied. The aim of this work is to increase the brush cytology diagnostic yield by elucidating the underlying mechanical phenomena. First, the mechanical interactions between the brush bristles and sampled tissue are analyzed. During brushing, mucus and detached cells are transferred to the space between the bristles through the capillary rise and flow eddies. These mass transfer mechanisms and their dependence on mucus rheology as a function of pH, brush displacement rate, and bristle geometry and configuration are examined. Lastly, results from *ex vivo* brushing experiments performed on porcine stomachs are presented. Clinical practitioners from a variety of disciplines can apply the findings of this study to outline clear procedures for cytological brushing to increase the sensitivity and specificity of the brushings.

## 1 Introduction

The diagnostic application of cytopathology covers virtually every part of the body. The branch of cytopathology, exfoliative cytology, examines cells scraped off or brushed from the surface of a tissue.^1,2^ Brush cytology is most commonly used for diagnosing esophagogastric and pancreaticobiliary diseases during gastrointestinal endoscopy.^3,4^ Use of brush cytology during an endoscopic retrograde cholangiopancreatography (ERCP) procedure for confirmatory diagnosis of biliary strictures can yield a wide range of results; for example, malignancy confirmed in 15–65% of cases of adenocarcinoma-associated biliary strictures and 44–80% of cases of cholangiocarcinoma.^5,6^ A study of over eight hundred patients with confirmed cancer reported a sensitivity of 42%, specificity of 98%, and positive predictive value of 98%.^6^ Overall, the diagnostic yield of ERCP-brush cytology is disappointingly low.^6^ The poor cellular volume of the sample is the main cause of this low yield. Multiple factors lead to this, including tumor cirrhosis (stiffness), small specimen sizes, and difficulty locating and targeting an abnormality, which results in inadequate tissue acquisition.^5^ Currently, the cellular yield of available cytology brushes does not differ significantly,^7^ but increasing the number of brushing passes will improve it.^8,9^

**Figure 1:**
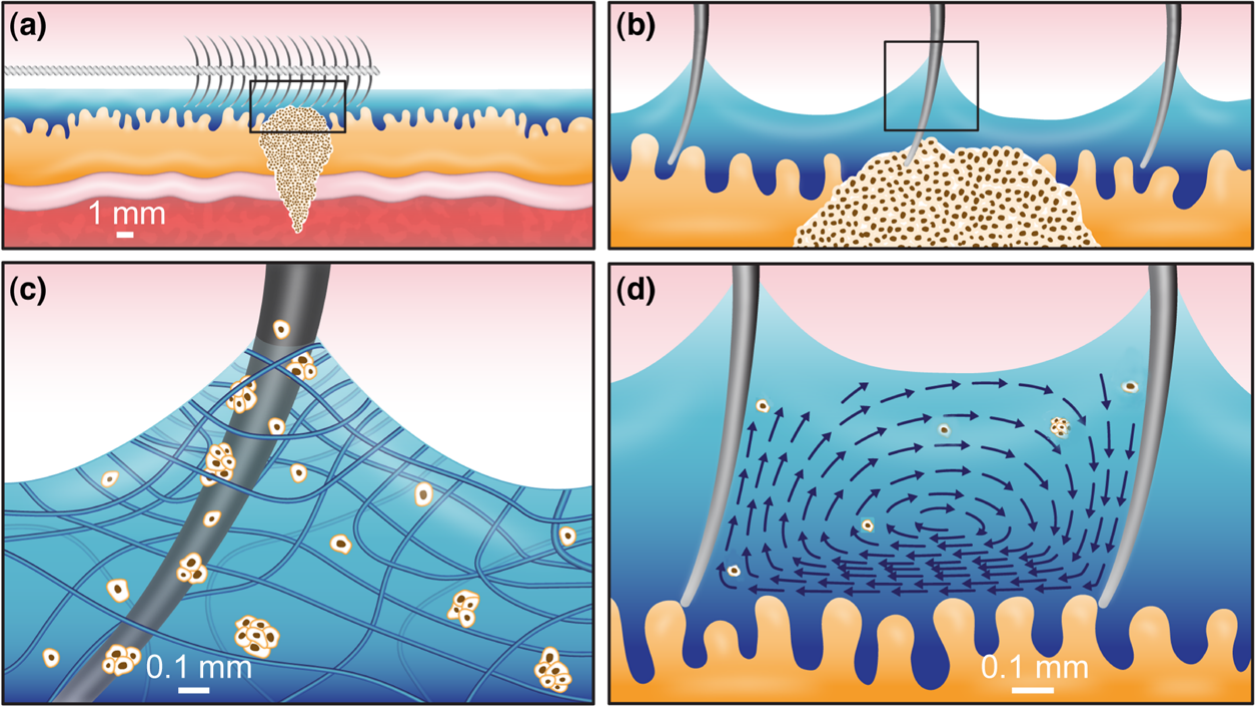
Overview of brush cytology. (a) Illustration of cytological brushing of malignant biliary strictures. (b) Close-up of the brush bristles in contact with the tumor during brushing. The mucus covers the lesion and is displaced due to capillary rise along the bristles. (c) Cellular debris from the lesion and mucin chains are primarily found on the mucous-coated bristle. (d) Illustration of a vortex formed in the “rectangular cavity” between bristles.

In general, cytology brushes are made up of nylon fiber bristles attached to thin metal shafts enclosed in a Teflon sheath. When an endoscope is used for ERCP, a brush emerges from its working channel. Afterward, the brush is moved back and forth across the lesion or stricture before being retracted and sent to cytopathology for analysis.^10^ Despite attempts to understand brush cytology and improve its diagnostic yield, there is no consensus to date regarding the biliary brushing parameters that would enable efficient sampling of suspicious lesions, such as the number of passes, brushing rate, and force applied. This study sought to better understand two mechanical phenomena occurring during brush cytology procedures, *i.e.*, shearing of the suspicious lesion during sampling and capture of tissue debris and cells that are expelled during sampling (see Figure 1). During sampling, bristles are submerged in the mucus, which covers the biliary epithelium. Bristles in contact with the mucosal surface shear the lesion as the brush is rubbed over it. As a result, the tissues are damaged, and cells or their debris are expelled into the surrounding mucus. The mucus wets the bristles, and a capillary rise occurs between them, moving the mucus upward. As brushing begins, a vortex forms within the space between the adjacent bristles. The mucus circulates between the bristles, entrapping and transporting expelled cells and tissue debris. The shear stress that develops on mucosal surfaces during brushing and their ability to shear epithelial cells is analyzed. In addition, the flow of mucus during brushing is studied, with a focus placed on capillary rise and vortices between bristles. Finally, *ex vivo* brush cytology experiments in porcine stomachs are presented.

## 2 Materials and Methods

### 2.1 Synthetic Mucus

Mucus solution was prepared following the procedure described by Huck et al.^11^ In brief, porcine stomach type II mucin (Sigma-Aldrich) was mixed with 0.9% w/w poly(acrylic acid) (PAA) (Carbopol 974P NF) and then 5% w/w was dissolved in distilled water. The solution was stirred at 300 rpm at 37 °C. The pH of the solution was then adjusted to 2, 4.9, and 7 using 1 M HCl/1 M NaOH. To visualize the mucus capillary rise, fluorescent beads were suspended in the mucus to a concentration of 0.05% v/v (Fluoro-Max Dyed Green Aqueous Fluorescent Particles, 9.9 μm, Thermo Fischer).

### 2.2 Brush Assembly

**Figure 2:**
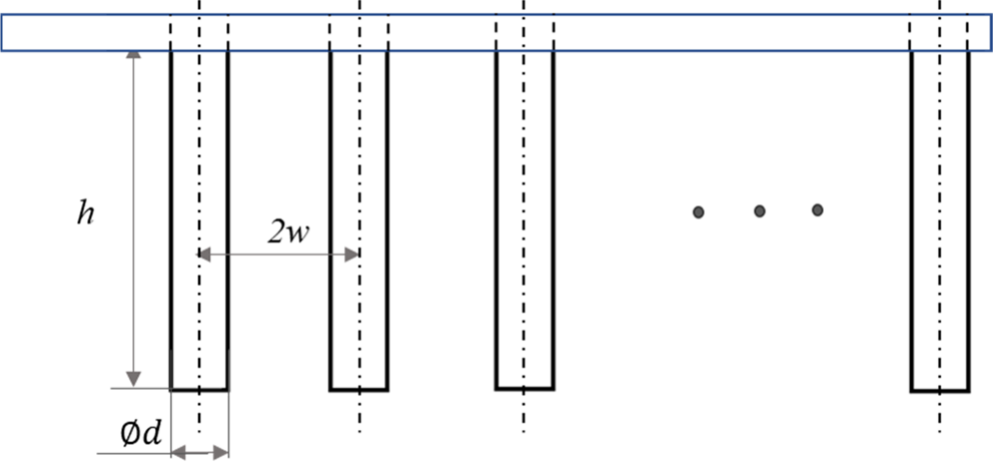
Brush model with an array of bristles. Each bristle has a length of *h* and a diameter of *d*, and the distance between their centerlines is 2*w*.

The brush’s body consists of a shaft and protruding bristles (see Figure 2). The shaft (8 × 8 × 2 mm^3^), which has an array of holes (diameter of 0.6 mm and depth of 1 mm), was fabricated by using a laser cutter on poly(methyl methacrylate) (PMMA) sheets. Bristles were made from Nylon-6,6 (polyamide 6,6)-based fibers (diameter, *d* = 0.16, or 0.45 mm) with an elastic modulus *E*_b_ = 2 GPa and Poisson’s ratio υ = 0.4. The fibers were inserted into the shaft’s holes and mounted using cyanoacrylate hydrophilic adhesive (3 M Scotch Super Glue). The protrusion height of the fibers after mechanical cutting was *h* = 1.32 mm, the distance between fiber axes was 2*w* = 1.08 mm, and the number of bristles per unit length was *N* = 1/2*dw*. Bristle assemblies with different configurations, and their effective radius, *R*_c_,^12^ are shown in Table 1. A commercial medical cytological brush (BCB-5–120–2-S, COOK Medical) was used as a control (Figure S1).

**Table 1:**
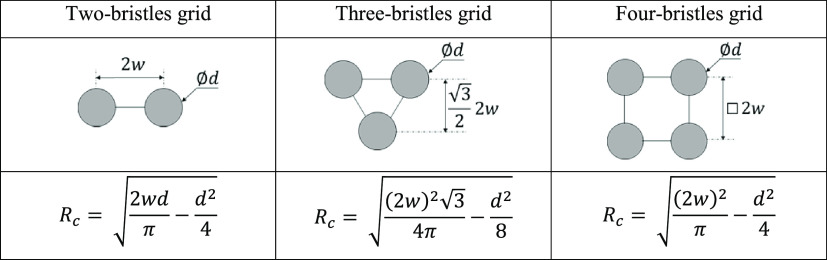
Assemblies of Bristles with Their Effective Radius, *R*_c_

### 2.3 Characterization Methods

#### 2.3.1 Rubbing Experimental System

An experimental system (Figure S1) was constructed to characterize the rubbing process. In this system, nylon bristles moved at a constant displacement rate while rubbing a clamped body and measuring the generated force with a load cell (Sensotec Instruments Load Cell model 31/1435–03, 1 N). The sample was clamped using a cyanoacrylate hydrophilic adhesive (3 M Scotch Super Glue) and kept in a distilled water bath until the experiment was conducted. The displacement rate was set to 1.58 mm/s, and the contact depth Δ-range was 0–0.4 mm. The brushing process was repeated 3 times for each sample, and the tests were conducted at room temperature. Images were captured with a Basler Ace acA2040–120 μm high-speed camera equipped with a Nikon AF-S VR Micro-NIKKOR 105 mm f/2.8G IF-ED lens.

#### 2.3.2 Rheological and Mechanical Characterization

A DHR-2 rotational rheometer (TA Instruments) was used to measure the shear viscosity of the mucin solutions. The measurements were performed using parallel plate geometry with a diameter of 40 mm and a 0.3 mm gap between the plates. A solvent trap cover for blocking the evaporation was used. The steady-state shear viscosity was measured using shear rates in the range of 10^–1^ – 10^3^ s^–1^ at 37 °C.

DMA (DMA Q800, TA Instruments, New Castle, DE) was used to measure the mechanical properties of mucosa disks (11.1 mm in diameter and 2.3 mm in height). The disks were punched out of the porcine stomach lining (see below Section 2.4) and subsequently compressed in strain-controlled mode at a strain rate of 2% min^–1^ at room temperature. Measurements were repeated 3 times for each sample.

#### 2.3.3 Contact Angle Measurements

Polyamide 6,6 (Sigma-Aldrich) was dissolved in 90% formic acid to a concentration of 0.1 g/mL. Borosilicate glass slides (2.5 × 2.5 cm^2^) were cleaned by sonication in an ultrasonic bath in acetone and then in water for 10 min each and then dried in an oven at 95 °C for 5 min. The polyamide 6,6 solution (0.5 mL) was then dropped on a borosilicate glass slide and spin-coated at a constant speed of 2000 rpm for 2 min. The films were allowed to dry at room temperature. After the films were placed in a homemade goniometer, a droplet of 10 μL mucus solution was deposited on each. Measurements were repeated 3 times for each sample.

### 2.4 *Ex Vivo* Experiments

Two porcine stomachs of 3-month-old pigs weighing ∼80 kg were sacrificed and then stored at 4 °C. Three longitudinal samples were cut. The dimensions of the test samples were 6 mm in width, 30 mm in length, and 4 mm in thickness. The tests were conducted at room temperature within 12 h of sacrifice. Rubbing, compression tests, and capillary rise experiments were conducted with the samples. All experimental protocols were approved by the Rambam Medical Center Ethics Committee (IL 0800522) and were carried out in accordance with the approved guidelines.

## 3 Results and Discussion

A simplified brush cytology model is presented below. First, the mechanical interaction between the bristles and the mucosal surface during brushing is analyzed. Mucus rheology is then discussed as a function of pH, and finally, capillary rise and flow eddies that form among bristles are discussed.

### 3.1 Brushing Mechanics

The mucosal lining of the stomach has tiny holes (∼30 μm in diameter in humans) called gastric pits,^13,14^ which are assumed to be periodically distributed along the mucosal surface. In addition, it is assumed that the mucosal surface is homogeneous and isotropic, with an elastic modulus *E*_m_ = 1–20 kPa and Poisson’s ratio υ = 0.4.^15,16^

The average stiffness of a consolidated tumor on the mucosal surface is in the range of *E*_t_ = 10–60 kPa with Poisson’s ratio υ = 0.3–0.4.^17−20^ The roughness of a mucosal surface can be approximated by a periodic function *f*(*x*) = *h*_p_ sin^2^(π*x*/*L*), where *h*_p_ is the asperity height, *h*_p_ ∼ 10 μm, and *L* is the period, *L* ∼ 150 μm.

As the brush is brought into contact with a mucosal surface, it is subjected to an external loading σ_*y*_. Assuming that the applied load does not vary along the *z*-axis, we can consider two-dimensional deformations independent of the out-of-plane direction. The condition of full contact of the bristles with a mucosal surface (the *x*–*z* plane) can be expressed as^21^
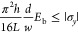
1

**Figure 3:**
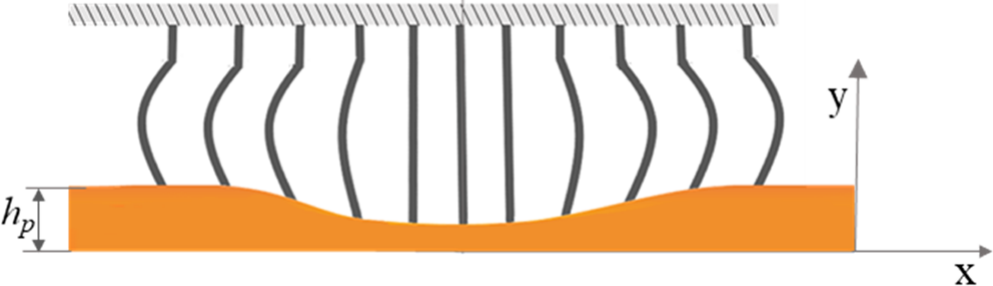
Brush loaded in compression against a mucosal surface (not to scale).

When the brush is uniformly displaced, the displacement in the *y*-direction, Δ_*y*_, is negative, corresponding to compressive stress, σ_*y*_ < 0 acting on the shaft of the brush, which stays stable (see Figure 3). Then, the resulting compressive force *F*_*y*_ acting on a bristle is *F*_*y*_ = σ_*y*_/*N* = 2*dw*σ_*y*_.

Above a certain threshold of compressive stress, the bristles may collapse. The critical buckling stress under the assumption that the bristle is clamped at the shaft of the brush and is simply supported at its contact with the surface, consistent with frictional restraints, would be approximately^22^
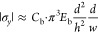
2where *C*_b_ = 10^–3^.

However, since the above expression is very sensitive to boundary conditions, it should be regarded as a scaling relationship.^21^

During brushing, the bristles interacting with the mucosal surface deform and detach biological cells from the extracellular matrix (ECM). The ECM is composed of proteins and polysaccharides secreted locally by the cells and fills the voids between cells.^23^ Deformation and detachment of cells are strongly influenced by the mechanical properties of the tumor, where prior works have shown a correlation between the mechanical properties of cells and pathophysiological states, such as cancer.^17,18^ As an example, the measured debonding forces obtained when trying to separate breast cells from their ECM were 211 ± 5 nN for normal cells and 133 ± 46 nN for cancer cells.^24^ The forces that developed during deformation and detachment of cultivated murine fibroblasts from one another were 350 ± 40 nN.^25^ In another work, the average detachment shear force of Chinese hamster ovary cells (CHO cells) from a glass substrate was estimated to be ∼400 nN and increased to ∼800 nN when the detachment rate was increased from 5 to 80 μm/s.^26^

**Figure 4:**
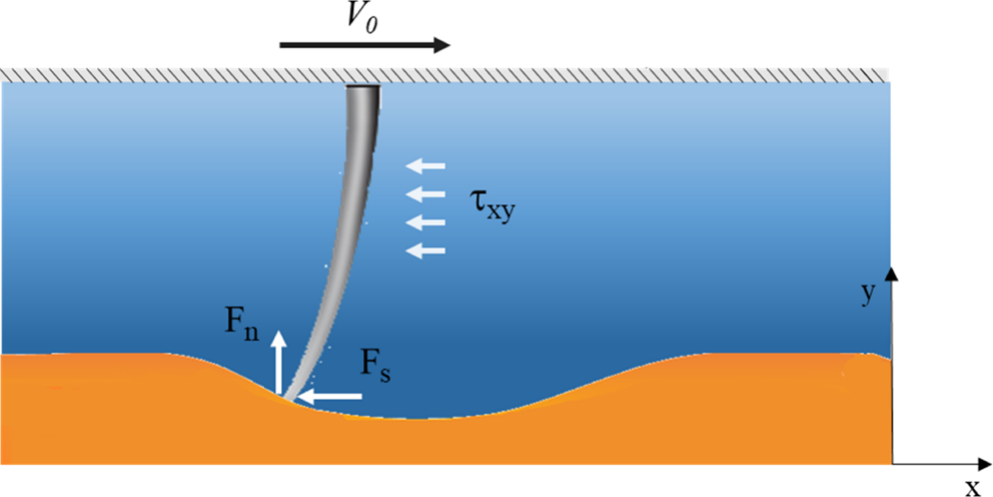
Single brush bristle moves at a constant velocity *V*_0_ when it is subjected to a normal force *F*_n_, shear force *F*_s_, and uniform shear stress τ_*xy*_ introduced by the mucus.

To estimate the shear force applied to the cells during rubbing, we developed a numerical three-dimensional (3D) model using the finite-element method (FEM) in COMSOL Multiphysics. In this model, we consider a single brush bristle (Nylon-6,6) with a length *h* = 1.32 mm and diameter *d* = 0.45 mm. The bristle is pressed upon a mucosal surface with a friction coefficient^16^ μ = 0.2 and then moved on top of it (see Figure 4). The brush shaft moves at a velocity *V*_0_ relative to the mucosal surface, resulting in the mucus motion that can be approximated as Couette flow.^27^ Then, the shear stress acting on the bristles is uniform τ_*xy*_ ∼ 10 Pa and the drag force acting on the bristle ends is ∼3 μN when considering *V*_0_ = 1 mm/s, *h* = 1 mm, *d* = 0.45 mm, and the mucus dynamic viscosity η = 10 Pa·s (assuming pH 2 is used).

**Figure 5:**
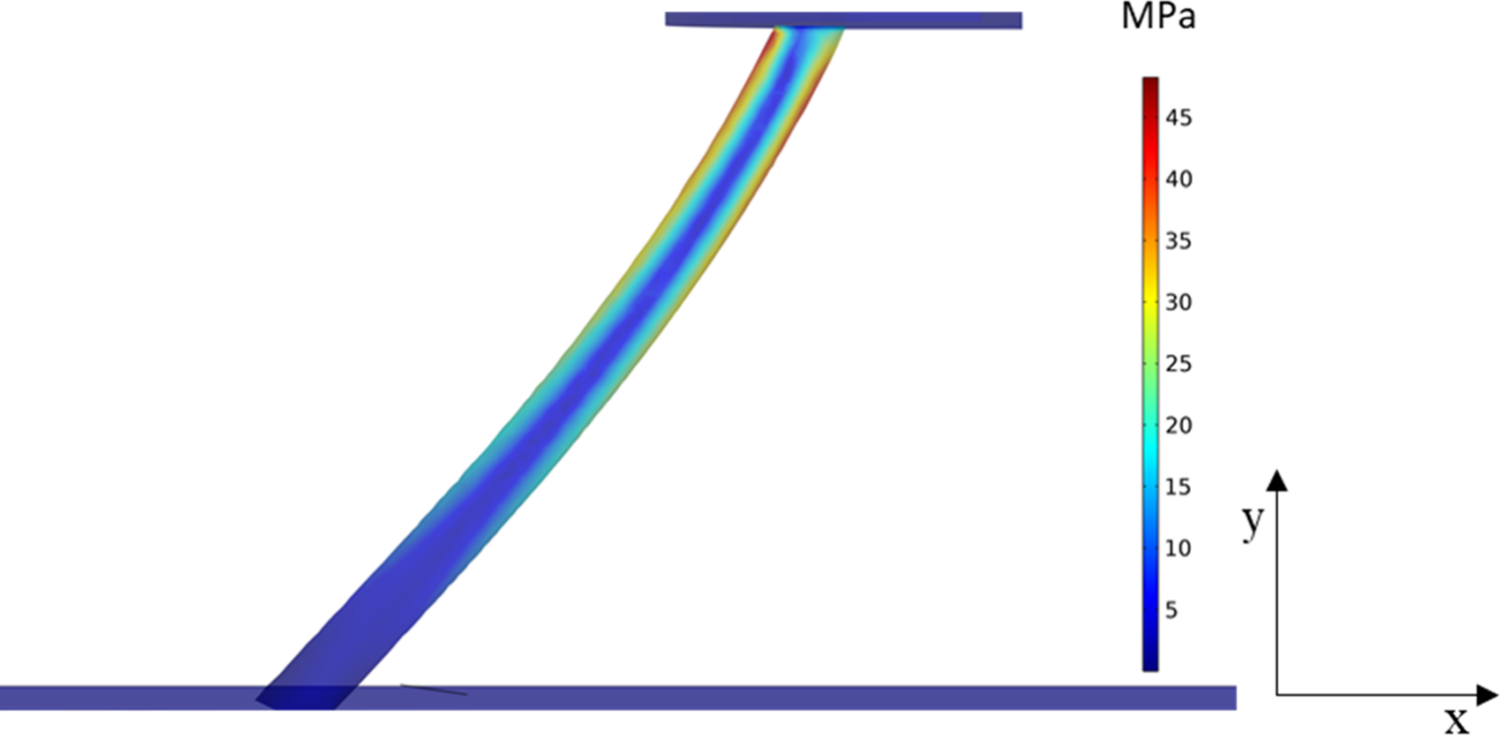
FEM simulation showing the stresses that develop during brushing at *V*_0_ = 1 mm/s for a single brush bristle rubbing a mucosal surface.

For example, after displacement Δ_*y*_ = −0.15 mm and preventing the penetration of the bristle into the surface, a maximal shear force *F*_s_ ∼ 1 mN was obtained (see Figure 5). As a comparison, the force required to detach a cell is approximately 4 orders of magnitude less.^24,26^ Once increasing the brush displacement, Δ_*y*_, the shear force increased as well. Clearly, due to the low shear velocity, the drag force applied by the mucus is negligible relative to the shear force.

### 3.2 Mucus Rheology

Gastrointestinal mucus primarily consists of water (∼95% w/w), mucins (∼0.2 to 5% w/v), globular proteins (∼0.5% w/v), salts (∼0.5 to 1% w/w), lipids (1–2% w/w), DNA, cells, and cellular debris.^28^ It is characterized by the formation of a continuous viscoelastic layer covering the mucosal surface with a thickness of 100–900 μm.^29^ The layer comprises a large number of physical entanglements stabilized by covalent and noncovalent interactions, including electrostatic, hydrophobic, hydrogen bonds, and other specific binding interactions that create a network and contribute to the mucus viscoelasticity.^29−31^ Mucins are charged polymers with high molecular weight (10–40 MDa) due to monomers consisting of glycosylated and nonglycosylated peptide blocks linked by intramolecular disulfide bridges.^32^ A mucin’s electrostatic character is determined by both the polypeptide backbone as well as the side chains of the oligosaccharide. The mucin chains are negatively charged at physiological pH, whereas, at very low pH, they are positively charged.^32^ While at physiological pH, mucus demonstrates a viscous behavior due to chain cross-linking, it exhibits an elastic behavior at acidic pH due to the extended conformation that yields mucus gelation in the stomach.^33,34^

**Figure 6:**
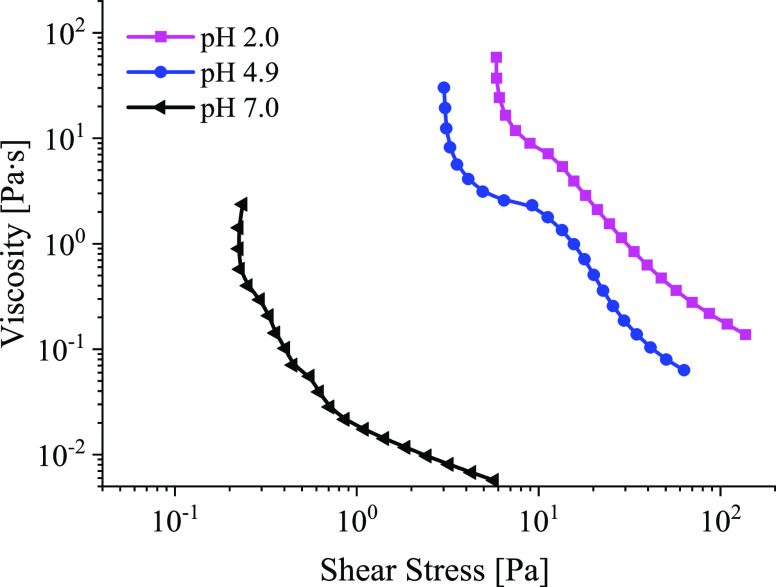
Flow curves of an artificial mucus solution with different pH values at 37 °C.

Viscosity measurements were conducted at pH 2, 4.9, and 7, simulating the viscosities of the mucus of the lumen of the stomach and submucosa interfaces; see Figure 6. In all mucus solutions, viscoplastic behavior was observed with pronounced yield stresses. Emulsions, concentrated suspensions, or polymer composites exhibit this behavior.^35^ The structure was destroyed when the yield stress was exceeded and the flow started. A change from pH 7 to pH 2 caused an increase in viscosity of 2 orders of magnitude on average. The yield stress, τ_*y*_, proved pH-dependent and was around 0.2, 2.5, and 5 Pa for pH of 2, 4.9, and 7, respectively. These results are consistent with Celli et al.^34^ The experimental data were fitted (see Table 2) to the Herschel–Bulkley model^36^
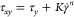
3where τ_*y*_ is the yield stress, *K* and *n* are the consistency and flow indices, respectively, and γ̇ is the shear rate that can be used to model the mucus flow curve.

**Table 2:** Rheological Parameters (Mean ± Standard Deviation) of the Herschel–Bulkley Model for Artificial Mucus Solutions (Measurements were Repeated 3 Times for Each Solution)

mucus solution	τ_*y*_ [Pa]	*K* [Pa·s^*n*^]	*n*
pH 2	5.07	3.85 ± 0.80	0.88 ± 0.10
pH 4.9	2.61	1.70 ± 0.43	0.90 ± 0.06
pH 7	0.23	0.06 ± 0.02	0.66 ± 0.04

The yield stress of mucus can be used to “tune” the shear rate introduced during brushing. The shear rate should exceed the yield stress-related rate before mucus networks are destroyed. For example, when working at pH 2, given that τ_*y*_ = 5 Pa and the respective viscosity η = 11 Pa·s, the shear rate where the mucus starts to flow is γ̇ = 0.63 s^–1^. Using the Couette flow approximation described above for a mucus layer with a thickness of 1 mm, the respective linear velocity of the brush is *V*_0_ = 0.63 mm/s. Once the mucus starts to flow, cells become trapped in the flow field between the bristles, which facilitates the collection of cells.

### 3.3 Capillary Rise

Capillary rise refers to a phenomenon, where a liquid is drawn into thin tubes or narrow gaps or channels between closely spaced micropillars or microstructures.^37,38^ This effect is driven by capillary forces, which arise from the intermolecular forces between the liquid molecules and solid surfaces. The height to which the liquid rises between the micropillars is determined by several factors, including the surface tension of the liquid, the wetting angle, the spacing between the pillars, the size and shape of the pillars, and gravity. As with micropillars, when a liquid wets a brush, a capillary rise may occur, allowing the fluid to be drawn into the bristles and further absorbed. Mucus is then evenly distributed between the bristles of a cytology brush when this occurs. Thus, if cells interact strongly with mucus, they will join the capillary rise and follow the mucus flow, resembling a flow of suspensions.^39^

**Figure 7:**
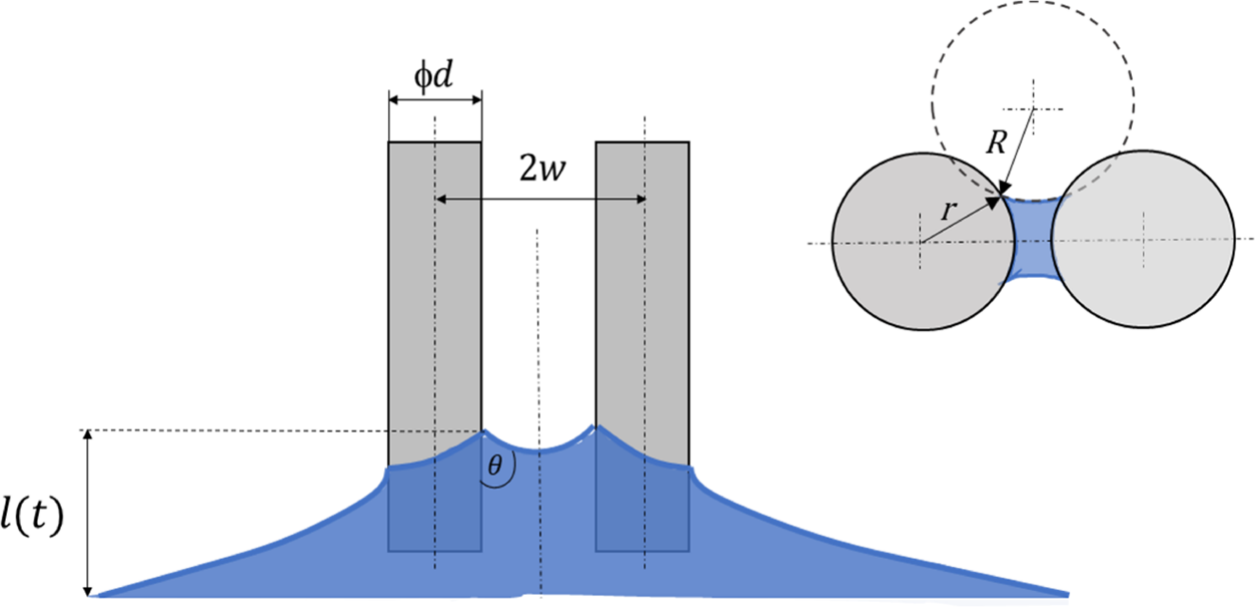
Schematic illustration of capillary rise between parallel cylinders with diameter *d* = 2*r* immersed in a liquid, where *l* is the maximal height of the meniscus, *R* is the meniscus radius of curvature in the horizontal plane just below the meniscus, and θ is the wetting angle.

To study the effect of the capillary rise in cytological brushes, we consider a simple model of the flow between two adjacent cylindrical bristles (see Figure 7). The bristles are rigid and thus are not affected by elastocapillary effects.^40^ After the bristles are immersed, the liquid rises, and the meniscus at the three-phase contact acquires a saddle shape and reaches an equilibrium height *l* above the horizontal liquid surface. The liquid is assumed to wet the bristles, and the contact angle θ is equal along the three-phase contact line. Since the meniscus dimensions are negligible in comparison to *l*, the liquid surface can be considered vertical between the bulk surface and the meniscus. Thus, one of the curvature radii of the surface is infinite, whereas the other is determined by radius *R* in the horizontal cross section.

The flow rate of liquid through a cylindrical tube due to capillary forces is typically determined by the Lucas–Washburn equation.^41^ It is derived from Hagen–Poiseuille’s law by neglecting inertial and gravitational forces to provide the dynamic capillary rising height as
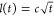
4where . The liquid properties are governed by the liquid surface tension γ, the wetting angle θ, and the dynamic viscosity η. The tube, in the case of closely spaced micropillars, or bristles, is characterized by an effective radius *R*_c_.^12^

**Figure 8:**
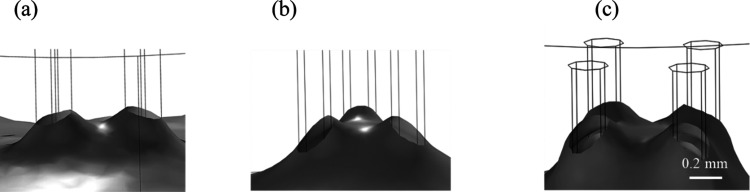
FEM simulation for the rising height of mucus on bristle unit cells. (a) Two-bristle unit cells with *R*_c_ = 0.23 mm, (b) triangular unit cell *R*_c_ = 0.36 mm, and (c) quadrilateral unit cell *R*_c_ = 0.55 mm. The wetting angle of the mucus^11^ at pH 4.9 is θ = 28° ± 2 (mean ± standard deviation). The viscosity is η ∼ 200 mPa·s, the density is ρ = 1017 kg m^–3^, and the surface tension is γ = 46 mN/m.^42^ The bristle diameter is *d* = 0.2 mm, and the distance between bristle centers is 2*w* = 1 mm.

**Figure 9:**
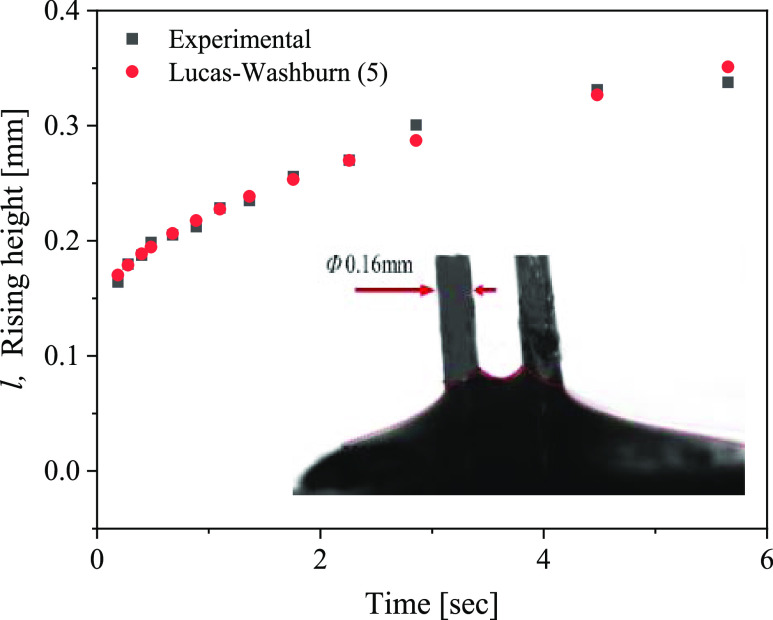
Time evolution of capillary rise between a two-bristle assembly that is immersed in a mucus reservoir (pH 4.9). Black squares represent the experimental results, and red dots represent the predictions of the Lucas–Washburn model (4). Inset: a typical image of capillary rise at a steady state.

The capillary rise of mucus has been studied by simulating the flow into bristle arrays with varying spacing in steady-state two-phase flow using COMSOL Multiphysics. Examples of capillary rise simulation using two-bristle unit cells and bristles arranged in quadrilaterals or triangle unit cells (see Table 1) are shown in Figure 8. The highest position of the meniscus was obtained with the quadrilateral unit cell, which is characterized by the largest *R*_c_. The experimental results of a two-bristle assembly immersed in mucus (pH 4.9) are shown in Figure 9. Comparing the experimental results and the prediction of the Lucas–Washburn model, we found that the best-fitted effective viscosity was η ∼ 200 mPa·s. Thus, based on the measured flow curve (Figure 6), the shear stress that developed during the brush immersion into the mucus was τ_*xy*_ ∼ 30 Pa.

**Figure 10:**
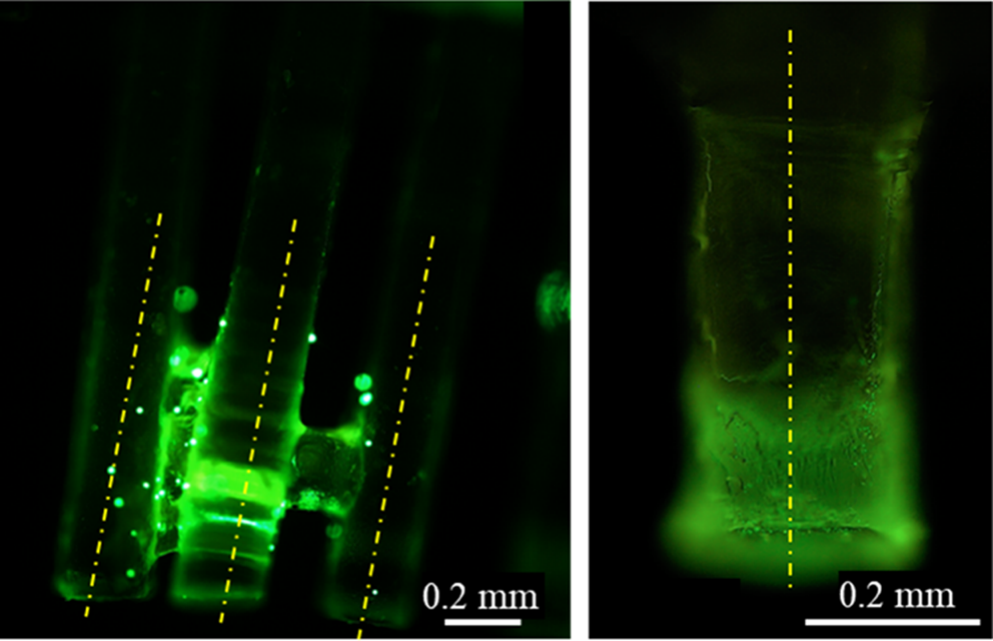
Mucus containing fluorescent beads illustrates cell entrapment between bristles and adsorption to them (yellow dash-dotted lines mark bristle axes). Images were taken immediately after mucus (pH 4.9) capillary rise. In both images, beads are seen distributed along the bristles. There is a small amount of mucus trapped between the bristles (left image).

To confirm the hypothesis regarding the entrapment of cells between bristles during the capillary rise, the bristles were immersed in mucus with suspended fluorescent beads (ϕ = 9.9 μm) at a concentration of 0.05% v/v and then viewed under a fluorescent microscope. After reaching the steady state of capillary rise, a visual inspection confirmed beads had accumulated on the bristles and between them (Figure 10).

### 3.4 Flow Modeling

**Figure 11:**
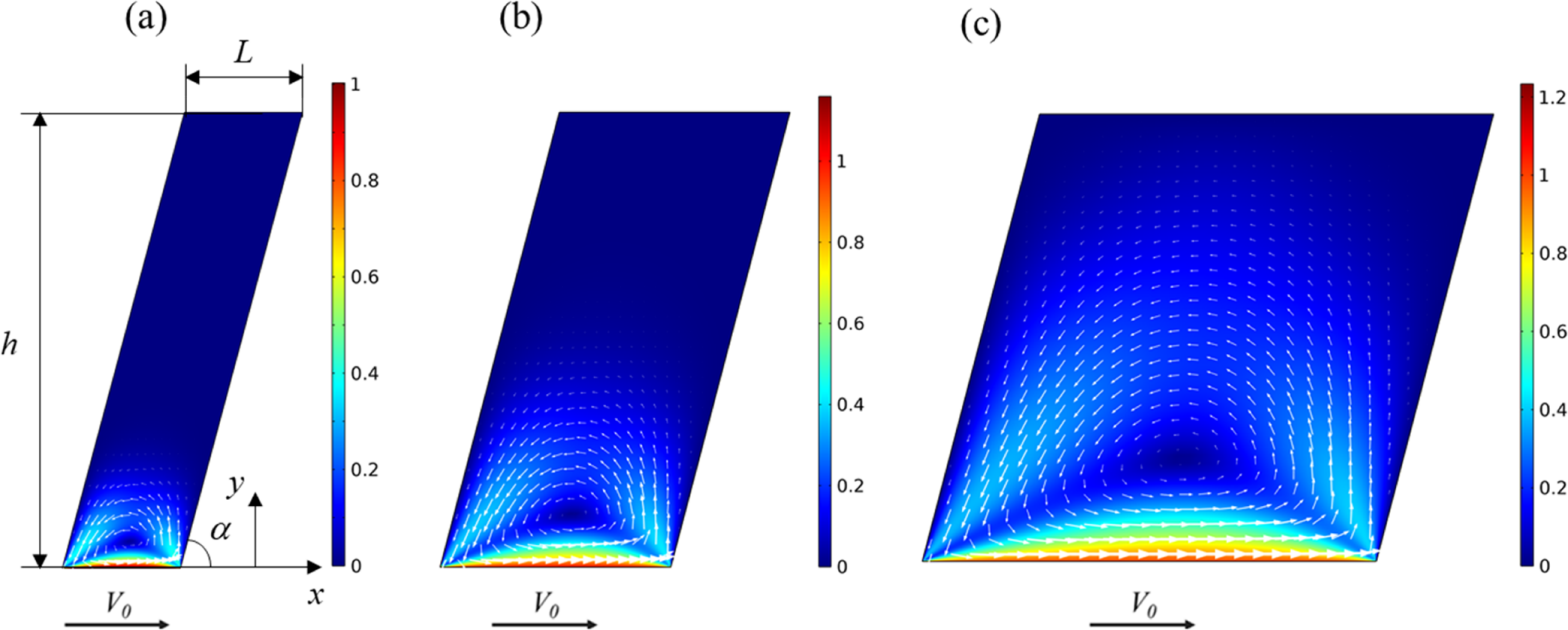
FEM simulation of lid-driven flow in a mucus-filled cavity between two adjacent bristles for different aspect ratios *L*/*h*. The color map represents the normalized magnitude of the velocity (5) and the white arrows represent the local scaled magnitude and direction of the velocity field. The lid velocity was *V*_0_ = 1 mm/s, and the height was *h* = 0.6 mm, while the width *L* varied as *L*/*h*: (a) 0.25, (b) 0.5, and (c) 1.

An investigation of mucus flow during brushing was conducted using a two-dimensional parallelogram cavity using COMSOL Multiphysics. The vertical walls of the cavity represent the parallel bristles, and the opened section is in contact with the brushed mucosal layer (Figure 11). The bristles are considered to be rigid and anchored to the mucosal surface. This ensures that the bristles do not bend or deform, nor do they move laterally as a result of fluid pressure. The motion generated in the cavity by the uniform translation corresponds to a lid-driven flow, as in the case of a steady flow involving closed streamlines.^43^ The parallelogram cavity is characterized by height *h*, width *L*, and angle α = 75° relative to the mucosal surface. The aspect ratio of the cavity is defined as *L*/*h*. The relative velocity (the lid velocity) between the brush and the mucosal surface was *V*_0_ = 1 mm/s, and the viscosity of the mucus was fixed to η ∼ 0.1 Pa·s. As a result, the Reynolds number *Re* = ρ*V*_0_*L*/η (ρ = 1017 kg m^–3^), representing the relative importance of inertia and viscous forces, was *Re* ≪ 1, i.e., viscous effects are dominant, yielding a creeping flow in the cavity.

In Figure 11, three configurations are shown with different aspect ratios of *L*/*h* = 0.25, 0.5, and 1. Here, the color map represents the normalized magnitude of the velocity, defined as
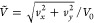
5where *v*_*x*_ and *v*_*y*_ are horizontal and vertical velocity components, respectively.

White arrows represent the local scaled magnitude and direction of the velocity field, *i.e.*, streamlines. It is evident that the streamlines describing the flow are nearly symmetric for all geometries and the Taylor scraper flow^44^ can be observed at the right corner, while the main vortex is formed in the middle of the cavity at different heights.

In a closed lid-cavity flow, satisfying the no-penetration of the fluid at the bristle surface results in the vertical velocity component *v*_*y*_, in addition to the horizontal velocity *v*_*x*_, induced by the lid velocity *V*_0_. The magnitude of the vertical velocity *v*_*y*_ increases as the aspect ratio *L*/*h* increases. Thus, the normalized magnitude of the velocity *Ṽ* also increases when changing *L*/*h* from 0.25 to 1 (see Figure 11). Furthermore, the increase in *v*_*y*_ and *L* results in the shift of the center of the vortex from 0.05*h* to 0.17*h* and 0.22*h* for *L*/*h* = 0.25, 0.5, and 1, respectively.

### 3.5 *Ex Vivo* Testing

**Figure 12:**
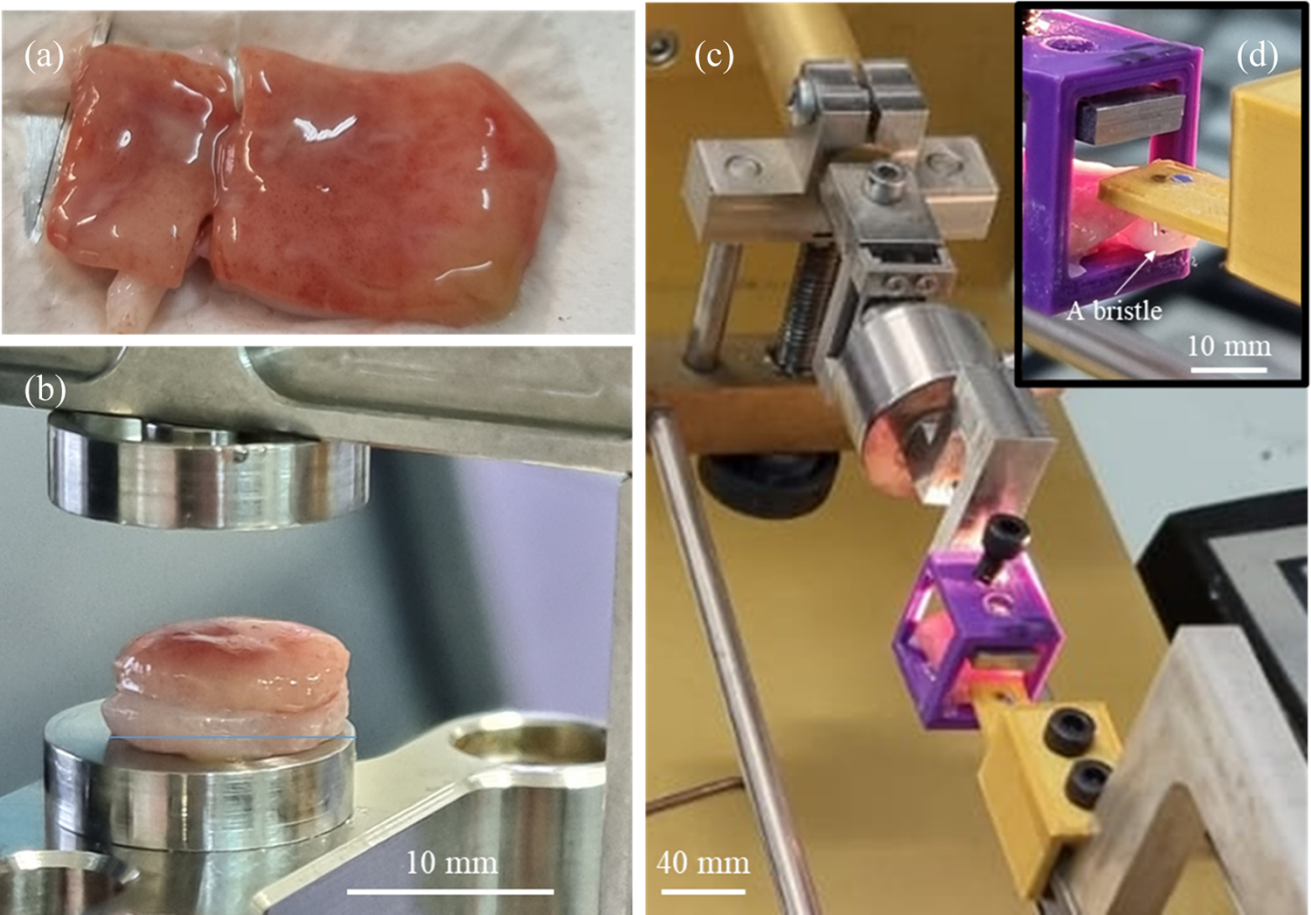
Experimental setup for mechanical characterization of the mucosa. (a) An image of a porcine stomach sample. (b) A mucosa disk is located between two plates in the compression jig. (c) The brushing setup that measures the shear force generated by bristles rubbing against the mucosa. (d) The assembly of a single Nylon-6,6 bristle (*d* = 0.45 mm, *h* = 0.45 mm).

**Figure 13:**
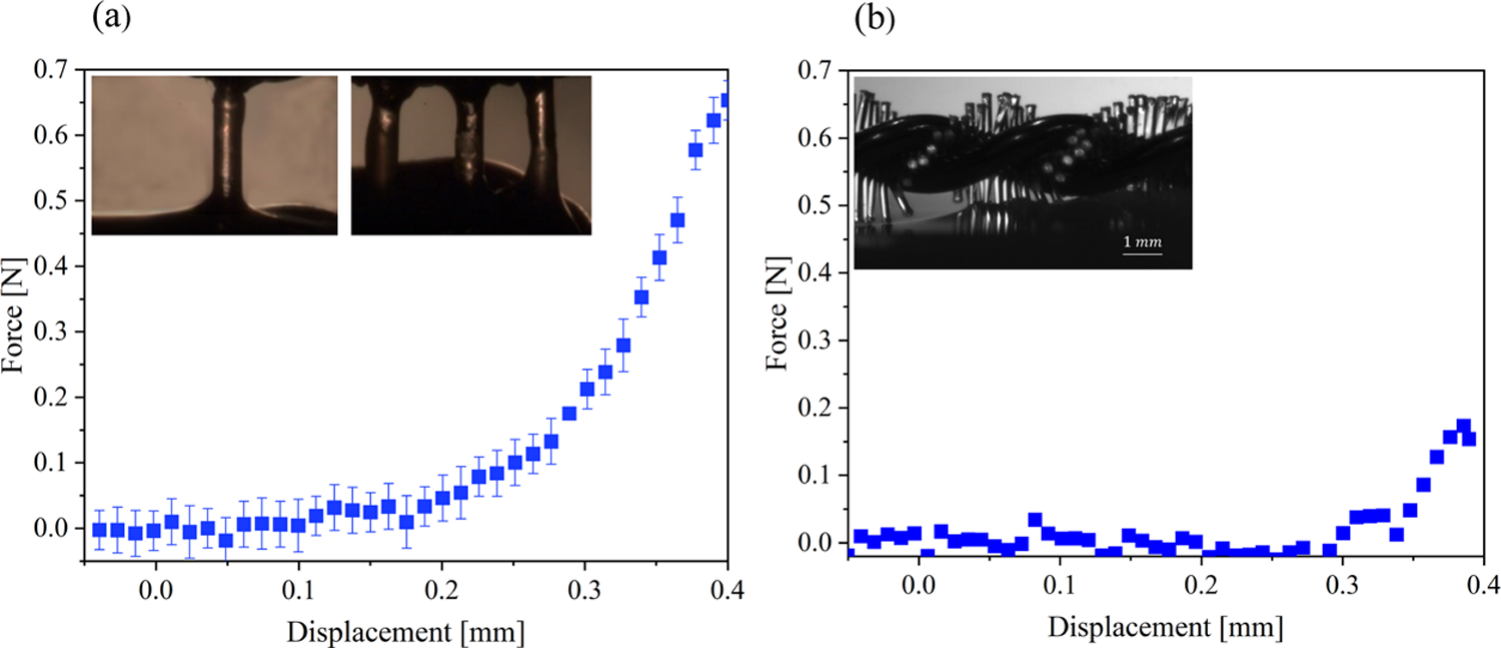
Force–displacement graphs measured during *ex vivo* brushing of stomach wall tissue with (a) a single bristle, where the force was measured using a single-bristle brush and a three-bristle brush setup (see the inset) with *d* = 0.45 mm, *h* = 0.45 mm, and 2*w* = 1.08 mm. (b) A medical cytological brush with *d* = 0.1 mm and *h* = 1 mm.

Cytological brushing experiments were conducted using intact porcine stomachs (Figure 12a). Samples were subjected to compression tests (Figure 12b), rubbing (Figure 12c,d), and capillary rise experiments. Through DMA compression tests, the elastic modulus was determined to be *E*_m_ = 2.6 ± 0.3 kPa (see Figure S2), in accordance with previous results.^15,16^ Considering isotropic linear elasticity and Poisson’s ratio ν = 0.4,^16^ the shear modulus is then *G* = *E*_m_/2(1 + ν) = 0.9 kPa. Accordingly, the developed shear stress is, for example, τ_*xy*_ = 10 Pa for a shear strain of γ_*xy*_ = 1.1%. The shear forces developed during rubbing against a mucosal surface were evaluated using a single-bristle, a three-bristle, and a medical cytology brush (Figure 13). The displacement rate of rubbing was set to 1.58 mm/s. When rubbing began, the shear force increased rapidly until it reached its maximum. Although it had nearly ten times as many bristles as the other brushes, the medical brush still had a lower maximal force of 0.18 N, apparently due to its thinner, less stiff bristles. However, the measured force value was nearly 2 orders of magnitude greater than the simulated force value *F*_s_ ∼ 1 mN. This deviation can be attributed to the experimental challenges associated with aligning the brush with the mucosal surface and folding of the surface, which were not considered in the simulation.

**Figure 14:**
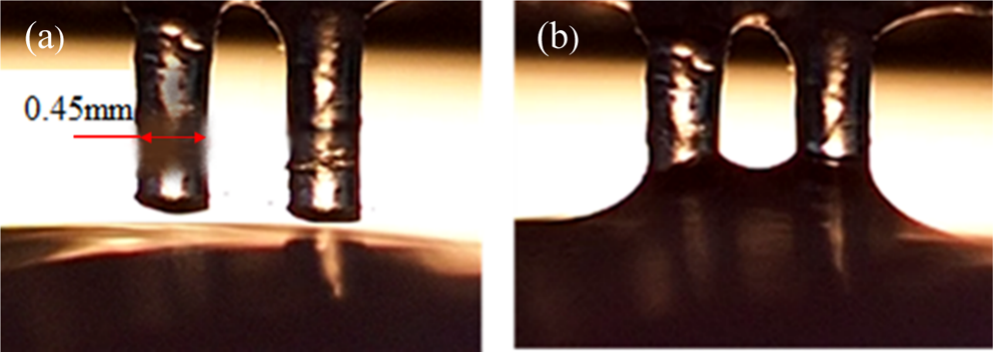
Capillary rise in a two-bristle assembly (*d* = 0.45 mm, *h* = 1.32 mm, and 2*w* = 1.08 mm) immersed in mucus during an *ex vivo* experiment. (a) Initial position above the mucosal surface. (b) Steady-state capillary rise showing fully developed radii and a substantial capillary rise from the initial position.

To replicate the acidic conditions in the stomach, the mucosal surface was wet (0.1 mL of 0.1 M PBS buffer adjusted to pH 4 by 1 M HCl) before evaluation of capillary rise. When the two-bristle brush was immersed, the capillary rise (4) was *l* = 0.1 ± 0.02 mm, which was in good agreement with the simulation results. Such a minor rise was expected due to the high viscosity of the mucus (η ∼ 8 Pa·s, see Figure 6) before yielding. However, after ten brushing passes, the capillary rise was more pronounced *l* = 0.47 ± 0.02 mm as the viscosity dropped by at least 2 orders of magnitude (see Figure 14).

## 4 Conclusions

This study aimed to increase the brush cytology diagnostic yield by investigating the mechanical interaction between the brush and the mucosal surface. To this end, a simple model of the mechanical interaction between bristles and mucosal and tumor surfaces was presented. It was discovered that mucus flowed between bristles through a capillary rise and lid-driven flow. A lid-driven flow was illustrated in a mucus-filled cavity (parallelogram) between two adjacent parallel bristles, where the relative velocity between the brush and the mucosal surface was taken as the lid velocity.

Mucus is described as one of the most rheologically complex fluids affected by pH. When a certain yield stress is exceeded during brushing, mucus begins to flow. The experimental and simulation results suggested that sheared cells detached from the lesion are transferred to the space between the bristles via capillary rises and eddies. Eddy development during brushing may play a significant role in cell entrapment. The eddies facilitate the mixing of the mucus and the detached cells, which follow the main vortex streamlines within the cavity. As the center of the vortex determines the extent of circulation, a higher vortex center, far from the mucosal surface, will extend the trapping region of the detached cells. According to the results, the center of the vortex will shift as the displacement rates and bristle spacing increase for a given length of bristles.

According to the results, the developed shear forces were ∼1.0 mN when the displacement rate of rubbing was ∼1.0 mm/s (∼1 s^–1^). In comparison, detaching a cell requires only a few orders of magnitude less force. Thus, the developed forces ensured proper cell sampling. Further, increasing the number of brushing passes had an effect on viscosity, which dropped by at least 2 orders of magnitude after ten passes.

The model developed in this study was validated in gastric brush cytology and can also be applied to brushing cytology in the gastrointestinal tracts, lungs, cervixes, and oral cavities. To enhance the brush cytology diagnostic yield, further research should be devoted to optimizing the cytological brush geometry and mechanical properties.

## Supporting Information Available

Additional experimental details that include the stress vs strain of the mucosa, and additional experimental details on the mechanical design of rubbing setup (PDF)

## References

[ref1] Baron T. H.; Lee J. G.; Wax T. D.; Schmitt C. M.; Cotton P. B.; Leung J. W. C. An in-vitro, randomized, prospective-study to maximize cellular yield during bile-duct brush cytology. Gastrointest. Endosc. 1994, 40 (2), 146–149. 10.1016/S0016-5107(94)70156-3. 8013811

[ref2] Marshall J. B.; Diazarias A. A.; Barthel J. S.; King P. D.; Butt J. H. Prospective evaluation of optimal number of biopsy specimen and brush cytology in the diagnosis of cancer of the colorectum. Am. J. Gastroenterol. 1993, 88 (9), 1352–1354. 8362828

[ref3] Boyd S.; Mustonen H.; Tenca A.; Jokelainen K.; Arola J.; Farkkila M. A. Surveillance of primary sclerosing cholangitis with ERC and brush cytology: risk factors for cholangiocarcinoma. Scand. J. Gastroenterol. 2017, 52 (2), 242–249. 10.1080/00365521.2016.1250281. 27806633

[ref4] Wilcox C. M.; Rodgers W.; Lazenby A. Prospective Comparison of Brush Cytology, Viral Culture, and Histology for the Diagnosis of Ulcerative Esophagitis in AIDS. Clin. Gastroenterol. Hepatol. 2004, 2 (7), 564–567. 10.1016/S1542-3565(04)00239-3. 15224280

[ref5] Fogel E. L.; DeBellis M.; McHenry L.; Watkins J. L.; Chappo J.; Cramer H.; Schmidt S.; Lazzell-Pannell L.; Sherman S.; Lehman G. A. Effectiveness of a new long cytology brush in the evaluation of malignant biliary obstruction: a prospective study. Gastrointest. Endosc. 2006, 63 (1), 71–77. 10.1016/j.gie.2005.08.039. 16377319

[ref6] de Bellis M.; Sherman S.; Fogel E. L.; Cramer H.; Chappo J.; McHenry L.; Watkins J. L.; Lehman G. A. Tissue sampling at ERCP in suspected malignant biliary strictures (Part 2). Gastrointest. Endosc. 2002, 56 (5), 720–730. 10.1016/S0016-5107(02)70123-5. 12397282

[ref7] Camp R.; Rutkowski M. A.; Atkison K.; Niedzwick L.; Vakil N. A prospective, randomized, blinded trial of cytological yield with disposable cytology brushes in upper gastrointestinal-tract lesions. Am. J. Gastroenterol. 1992, 87 (10), 1439–1442. 1415101

[ref8] Barkun A.; Liu J.; Carpenter S.; Chotiprasidhi P.; Chuttani R.; Ginsberg G.; Hussain N.; Silverman W.; Taitelbaum G.; Petersen B. T.; et al. Update on endoscopic tissue sampling devices. Gastrointest. Endosc. 2006, 63 (6), 741–745. 10.1016/j.gie.2006.02.041. 16650530

[ref9] Wang J. J.; Xia M. X.; Jin Y. B.; Zheng H. M.; Shen Z. Y.; Dai W. M.; Li X. M.; Kang M.; Wan R.; Lu L. G.; Hu B.; Wan X. J.; Cai X. B. More Endoscopy-Based Brushing Passes Improve the Detection of Malignant Biliary Strictures: A Multicenter Randomized Controlled Trial. Am. J. Gastroenterol. 2022, 117 (5), 733–739. 10.14309/ajg.0000000000001666. 35108222

[ref10] Juvvala K.; Bajwa M.; Bank L.; Marhaba A. Brush Cytology During ERCP for the Diagnosis of Biliary and Pancreatic Malignancies. Am. J. Gastroenterol. 2018, 113, S1517.

[ref11] Huck B. C.; Hartwig O.; Biehl A.; Schwarzkopf K.; Wagner C.; Loretz B.; Murgia X.; Lehr C. M. Macro- and Microrheological Properties of Mucus Surrogates in Comparison to Native Intestinal and Pulmonary Mucus. Biomacromolecules 2019, 20 (9), 3504–3512. 10.1021/acs.biomac.9b00780. 31419118

[ref12] Princen H. M. Capillary phenomena in assemblies of parallel cylinders. I. capillary rise between 2 cylinders. J. Colloid Interface Sci. 1969, 30 (1), 69–75. 10.1016/0021-9797(69)90379-8.

[ref13] Zhang J. N.; Li Y. Q.; Zhao Y. A.; Yu T.; Zhang J. P.; Guo Y. T.; Liu H. Classification of gastric pit patterns by confocal endomicroscopy. Gastrointest. Endosc. 2008, 67 (6), 843–853. 10.1016/j.gie.2008.01.036. 18440377

[ref14] Altikriti M.; Albagdadi F.; Henry R. W.; Hoskins J.; Titkemeyer C.; Strain G. The normal structure of regional feline gastric mucosae - scanning electron-microscopic study. Scanning Microsc. 1987, 1 (4), 1871–1880. 3433068

[ref15] Dargar S.; Rahul; Kruger U.; De S. In Vivo Layer-Specific Mechanical Characterization of Porcine Stomach Tissue Using a Customized Ultrasound Elastography System. J. Biomech. Eng. 2019, 141 (10), 101004 10.1115/1.4043259. 30901383 PMC6808004

[ref16] Chen J. N.; Suenaga H.; Hogg M.; Li W.; Swain M.; Li Q. Determination of oral mucosal Poisson’s ratio and coefficient of friction from in-vivo contact pressure measurements. Comput. Methods Biomech. Biomed. Eng. 2016, 19 (4), 357–365. 10.1080/10255842.2015.1028925. 26024011

[ref17] Alibert C.; Goud B.; Manneville J. B. Are cancer cells really softer than normal cells?. Biol. Cell 2017, 109 (5), 167–189. 10.1111/boc.201600078. 28244605

[ref18] Blanco B.; Gomez H.; Melchor J.; Palma R.; Soler J.; Rus G. Mechanotransduction in tumor dynamics modeling. Phys. Life Rev. 2023, 44, 279–301. 10.1016/j.plrev.2023.01.017. 36841159

[ref19] Islam M. T.; Tang S. Y.; Liverani C.; Saha S.; Tasciotti E.; Righetti R. Non-invasive imaging of Young’s modulus and Poisson’s ratio in cancers in vivo. Sci. Rep. 2020, 10 (1), 7266 10.1038/s41598-020-64162-6. 32350327 PMC7190860

[ref20] Masuzaki R.; Tateishi R.; Yoshida H.; Sato T.; Ohki T.; Goto T.; Yoshida H.; Sato S.; Sugioka Y.; Ikeda H.; Shiina S.; Kawabe T.; Omata M. Assessing liver tumor stiffness by transient elastography. Hepatol. Int. 2007, 1 (3), 394–397. 10.1007/s12072-007-9012-7. 19669335 PMC2716830

[ref21] Hui C. Y.; Jagota A.; Lin Y. Y.; Kramer E. J. Constraints on microcontact printing imposed by stamp deformation. Langmuir 2002, 18 (4), 1394–1407. 10.1021/la0113567.

[ref22] Timoshenko S. P.; Gere J. M. Theory of Elastic Stability, 2nd ed.; McGraw-Hill: New York, 1961.

[ref23] Bauer M.; Morales-Orcajo E.; Klemm L.; Seydewitz R.; Fiebach V.; Siebert T.; Böl M. Biomechanical and microstructural characterisation of the porcine stomach wall: Location- and layer-dependent investigations. Acta Biomater. 2020, 102, 83–99. 10.1016/j.actbio.2019.11.038. 31760221

[ref24] Ani C. J.; Obayemi J. D.; Uzonwanne V. O.; Danyuo Y.; Odusanya O. S.; Hu J.; Malatesta K.; Soboyejo W. O. A shear assay study of single normal/breast cancer cell deformation and detachment from poly-di-methyl-siloxane (PDMS) surfaces. J. Mech. Behav. Biomed. Mater. 2019, 91, 76–90. 10.1016/j.jmbbm.2018.11.012. 30544025

[ref25] Yamamoto A.; Mishima S.; Maruyama N.; Sumita M. A new technique for direct measurement of the shear force necessary to detach a cell from a material. Biomaterials 1998, 19 (7–9), 871–879. 10.1016/S0142-9612(97)00248-2. 9663764

[ref26] Hashimoto S.; Adachi M.; Iwata F. Investigation of shear force of a single adhesion cell using a self-sensitive cantilever and fluorescence microscopy. Jpn. J. Appl. Phys. 2015, 54 (8), 08LB03 10.7567/JJAP.54.08LB03.

[ref27] Batchelor G. K. An Introduction to Fluid Dynamics; Cambridge University Press, 1967.

[ref28] Leal J.; Smyth H. D. C.; Ghosh D. Physicochemical properties of mucus and their impact on transmucosal drug delivery. Int. J. Pharm. 2017, 532 (1), 555–572. 10.1016/j.ijpharm.2017.09.018. 28917986 PMC5744044

[ref29] Wagner C. E.; Turner B. S.; Rubinstein M.; McKinley G. H.; Ribbeck K. A Rheological Study of the Association and Dynamics of MUC5AC Gels. Biomacromolecules 2017, 18 (11), 3654–3664. 10.1021/acs.biomac.7b00809. 28903557 PMC5776034

[ref30] Bansil R.; Turner B. S. The biology of mucus: Composition, synthesis and organization. Adv. Drug Delivery Rev. 2018, 124, 3–15. 10.1016/j.addr.2017.09.023. 28970050

[ref31] Lai S. K.; Wang Y. Y.; Wirtz D.; Hanes J. Micro- and macrorheology of mucus. Adv. Drug Delivery Rev. 2009, 61 (2), 86–100. 10.1016/j.addr.2008.09.012. PMC2736374 19166889

[ref32] Nyström B.; Kjoniksen A. L.; Beheshti N.; Maleki A.; Zhu K. Z.; Knudsen K. D.; Pamies R.; Cifre J. G. H.; de la Torre J. G. Characterization of polyelectrolyte features in polysaccharide systems and mucin. Adv. Colloid Interface Sci. 2010, 158 (1–2), 108–118. 10.1016/j.cis.2009.05.003. 19482258

[ref33] Parlato R. M.; Greco F.; Maffettone P. L.; Larobina D. Effect of pH on the viscoelastic properties of pig gastric mucus. J. Mech. Behav. Biomed. Mater. 2019, 98, 195–199. 10.1016/j.jmbbm.2019.06.008. 31254906

[ref34] Celli J. P.; Turner B. S.; Afdhal N. H.; Ewoldt R. H.; McKinley G. H.; Bansil R.; Erramilli S. Rheology of gastric mucin exhibits a pH-dependent sol-gel transition. Biomacromolecules 2007, 8 (5), 1580–1586. 10.1021/bm0609691. 17402780

[ref35] Malkin A. Y.; Isayev A. I. Rheology Concepts, Methods, and Applications; ChemTec: Toronto, 2006.

[ref36] Macosko C. W. Rheology Principles, Measurements and Applications; Wiley-VCH: Canada, 1994.

[ref37] Delannoy J.; Lafon S.; Koga Y.; Reyssat É.; Quéré D. The dual role of viscosity in capillary rise. Soft Matter 2019, 15 (13), 2757–2761. 10.1039/C8SM02485E. 30693361

[ref38] Liu Z.; He X. C.; Han J. X.; Zhang X. H.; Li F.; Li A.; Qu Z. G.; Xu F. Liquid wicking behavior in paper-like materials: mathematical models and their emerging biomedical applications. Microfluid. Nanofluid. 2018, 22 (11), 132 10.1007/s10404-018-2151-4.

[ref39] Jeong D. H.; Lee M. K. H.; Thiévenaz V.; Bazant M. Z.; Sauret A. Dip coating of bidisperse particulate suspensions. J. Fluid Mech. 2022, 936, A36 10.1017/jfm.2022.79.

[ref40] Gat A. D.; Gharib M. Elasto-capillary coalescence of multiple parallel sheets. J. Fluid Mech. 2013, 723, 692–705. 10.1017/jfm.2013.86.

[ref41] Washburn E. W. The Dynamics of Capillary Flow. Phys. Rev. 1921, 17 (3), 273 10.1103/PhysRev.17.273.

[ref42] Mikos A. G.; Peppas N. A. Measurement of the surface-tension of mucin solutions. Int. J. Pharm. 1989, 53 (1), 1–5. 10.1016/0378-5173(89)90354-2.

[ref43] Pan F.; Acrivos A. Steady flows in rectangular cavities. J. Fluid Mech. 1967, 28 (4), 643–655. 10.1017/S002211206700237X.

[ref44] Taylor G. I. On Scraping Viscous Fluid from a Plane Surface. In Miszellaneen der angewandten Mechanik; Tollmien W., Ed.; De Gruyter: Berlin, 1962.

