# Supplementary material for: Mechanical Perspective on Increasing Brush Cytology Yield
Source: ACS Biomater Sci Eng. 2024 Feb 19;10(3):1743–52. doi: 10.1021/acsbiomaterials.3c00935 (PMC10934267; doi:10.1021/acsbiomaterials.3c00935)
Supplement: Supplementary file 1 — ab3c00935_si_001.pdf [file ab3c00935_si_001.pdf]

Mechanical Perspective on Increasing Brush Cytology Yield

Supporting Information

Iyad Khamaysi,[1,2,*] Ronen Firman,[3] Patrick Martin,[3] Gleb Vasilyev,[3]
Evgeniy Boyko,[3] Eyal Zussman[3,*]

[1]Department of Gastroenterology, The Ruth and Bruce Rappaport Faculty of Medicine, Technion – Israel Institute of Technology, Haifa 3525433, Israel
[2]Gastroenterology Institute, Rambam Health Care Campus, Haifa 3109601, Israel
[3]Faculty of Mechanical Engineering, Technion – Israel Institute of Technology, Haifa 3200003, Israel

An illustration of the rubbing setup is presented in Fig. S1. The rubbing force was measured with a load cell (Sensotec Instruments Load Cell model 31/1435-03, 1 N). The displacement rate was set to 1.58 *mm/s* and the contact depths Δ range was 0-0.4 *mm*.

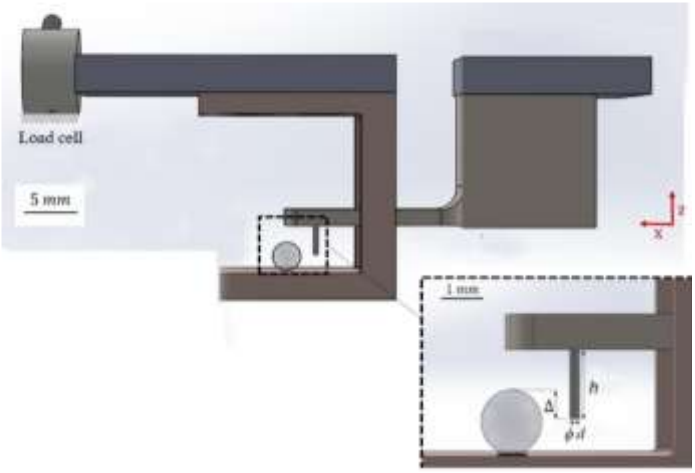

Figure S1: Experimental setup for rubbing experiment. The inset shows a brush with bristle brushing a spherical sample.

The stomach bilayer was cut to fit the size of the plates with a punch. The compressive modulus was determined using DMA Q800, see Fig. S2.

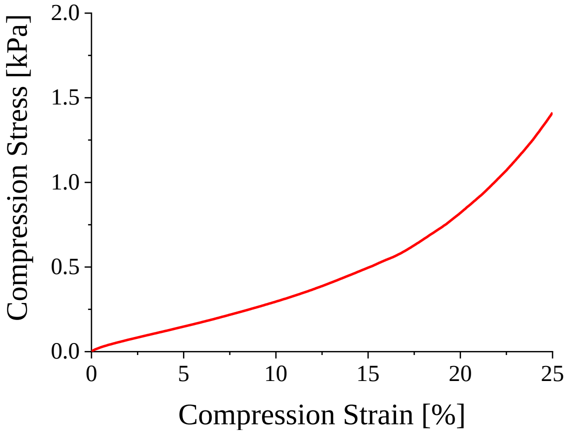

Figure S2: Stress vs. strain of the mucosa. The determined compression modulus is $E_m = 2.6 \pm 0.3\ kPa$.
